# Frequency and characteristics of emphysema in adults with *FLNA* variants: a single-center study

**DOI:** 10.1186/s13023-025-04068-6

**Published:** 2025-11-05

**Authors:** Arthur Michalski, Catherine Vincent-Delorme, Silvia Demoulin-Alexikova, Thomas Smol, Paul Felloni, Olivier Le Rouzic, Thierry Perez, Nathalie Bautin, Lidwine Wémeau, Malika Balduyck, Farid Zerimech, François Pontana, Pascal Delsart, Emeline Cailliau, Cécile Chenivesse, Victor Valentin

**Affiliations:** 1 University Lille, CHU Lille, Lille, F-59000 France; 2 Service de Génétique Clinique Guy Fontaine, CHU Lille, Lille, F-59000 France; 3 University Lille, CNRS, Inserm, CHU Lille, Institut Pasteur Lille, U1019 – UMR 9017 – CIIL – Center for Infection and Immunity of Lille, Centre de référence constitutif des maladies pulmonaires rares Orphalung, Lille, F-59000 France; 4 University Lille, CHU Lille, ULR7364 – RADEME – Maladies Rares du Développement embryonnaire et du Métabolisme, Lille, F-59000 France; 5 Département d’Imagerie Thoracique, University Lille, CHU Lille, Lille, F-59000 France; 6 Service de Biochimie HMNO, CHU Lille, University Lille, ULR 7364 - RADEME, ULR 4483 - IMPECS, Lille, France; 7 University Lille, CHU Lille, Institut Coeur-Poumon, Lille, F-59000 France; 8 Departement de Biostatistiques, CHU Lille, Lille, F-59000 France

**Keywords:** Emphysema, *FLNA* mutation, Dyspnea

## Abstract

**Introduction:**

*FLNA* pathogenic variants lead to various congenital malformations. Pulmonary manifestations were reported in early age. In adults, few cases of emphysema were published. We aimed to assess the frequency and characteristics of emphysema in adults with a pathogenic or probably pathogenic variant in *FLNA*.

**Method:**

We conducted a transversal single-center study. Adults with *FLNA* pathogenic variant followed in Lille University Hospital were identified among the French rare disease database BaMaRa^®^. They attended medical examination, non-contrast chest CT-scan and pulmonary function tests (PFT). We collected medical history, tobacco smoking status, occupational and domestic exposures. When emphysema was identified, the PTPN6 gene was sequenced and alpha1antitrypsin deficiency searched. The primary objective of the study was to assess the frequency of emphysema. Secondary objectives were to evaluate emphysema type, distribution and extension, lung function in case of emphysema and the frequency of unexplained emphysema.

**Results:**

Among the 29 patients identified, 10 met non-inclusion criteria, 4 declined, 5 could not be reached and so 10 patients were included. The frequency of emphysema was 70%. It was most often centrilobular and located in upper lobes (*n* = 4) with a median volume of 8.8% (IQR: 3.0–18.0%) of the total lung volume. The frequency of unexplained emphysema was estimated to be 57.1% of emphysema cases. PFT revealed airflow limitation (*n* = 3), reduced DLCO (*n* = 5) and small airway disease on IOS (*n* = 5).

**Conclusion:**

These preliminary results suggest that adults with *FLNA* pathogenic variant frequently develop emphysema, allowing for a larger study to be considered.

**Trial registration:**

ClinicalTrials.gov, NCT05550844, Registered 19 September 2022 https://clinicaltrials.gov/study/NCT05550844?cond=FLNA&rank=1.

To the Editor,

The X-linked filamin A gene *(FLNA)* encodes for filamin-A, an actin-binding protein playing a key role in the cytoskeleton organization. Loss-of-function pathogenic variants lead to periventricular nodular heterotopia (PVNH) and various gastro-intestinal, cardiovascular and connective tissue disorders while gain-of-function pathogenic variants result in otopalatodigital syndrome spectrum disorders. A range of pulmonary manifestations including neonatal respiratory failure, congenital emphysema, interstitial lung disease, atelectasis and recurrent infections have been reported in early age [1]. In adults, only few cases of emphysema have since been published [2–4]. We recently described two cases of emphysema within the same family unexplained by environmental exposure in adult women with *FLNA* loss-of-function pathogenic variants [5]. Since, three additional cases were described in women [3]. However, the link between *FLNA*-pathogenic variants and emphysema is unknown. In this preliminary study, we aimed to assess the frequency and characteristics of emphysema in adults with a pathogenic variant in FLNA.

We conducted a transversal single-center prospective study. All adults (≥ 18 years) with *FLNA* pathogenic or probably pathogenic variants followed in Lille University Hospital were identified in the French rare diseases database BaMaRa^®^ and proposed to participate in the study. We excluded patients unable to give consent or under guardianship and pregnant or breastfeeding women. All participants provided written informed consent. The study was approved by the Institutional Review Board Ouest II of Angers University Hospital (2022-A00972-41) and was declared to the National Commission on Informatics and Liberty.

Participants attended medical examination, non-contrast chest CT-scan and pulmonary function tests (PFT). We collected socio-demographic data, medical history, tobacco smoking status, occupational and domestic exposures with a focus on wood dust, fumes and pollution defined as a high traffic road at less than 500 m from home. Passive smoking was defined as the presence of a smoker within the home. A chest radiologist performed qualitative and quantitative analysis of emphysema by visual and automatized computed quantification of the number of voxels with density less than − 950HU (Syngo.via^®^, Siemens HealthinnersⓇ). PFT included spirometry, plethysmography, diffusion capacity for carbon monoxide (DLCO) and impulse oscillometry (IOS^®^, Jaeger). Global lung Initiative (GLI) predicted values were used for spirometry, volumes and DLCO and those from Oostveen et al. for IOS [6]. Forced expiratory volume in 1 s (FEV1), forced vital capacity (FVC), FEV1/FVC, forced expiratory flow between 25 and 75% of FVC (FEF25-75) and DLCO values were considered abnormal when the z-score was below − 1.64. Residual volume (RV), total lung capacity (TLC), RV/TLC, resistance at 5 Hz (R5), reactance at 5 Hz (X5) and integrated area of low frequency reactance (AX) values were considered abnormal when the z-score was over 1.64. Airflow limitation was defined by a FEV1/FVC < lower limit normal and lung hyperinflation by a RV/TLC >upper limit normal. The difference R5-R20 was considered abnormal when higher than 0.07 kPa/L/s, then defining small airway dysfunction (SAD).

When emphysema was identified, the *PTPN6* gene [7] was sequenced using Sanger method (ABI Prism 3730XL Genetic Analyzer; Applied Biosystems^®^) and alpha-1-antitrypsin (AAT) deficiency was searched (serum level, isoelectric focusing and gene sequencing).

The primary objective was to assess the frequency of emphysema, defined as the presence of focal areas or regions of low attenuation, usually without visible walls. Secondary objectives were to evaluate (1) emphysema type (centrilobular, panlobular or paraseptal), distribution (upper, lower lobes, or no predominance) and extension, (2) lung function in the whole population and in participants with emphysema and (3) the frequency of unexplained emphysema, defined as low tobacco exposure (active smoking < 5 pack-years and no significant passive smoking), no exposure to noxious particles, no AAT deficiency and no pathogenic variant in *PTPN6* gene. Our approach was specifically designed based on the study by Bossé et al. [7], which identified a heterozygous missense variant (Ala455Thr) within a critical functional site of the PTPN6 protein. Accordingly, we focused our investigation on point mutations within this functionally important region, particularly residues 453 to 459, using targeted Sanger sequencing.

Twenty-eight patients with *FLNA* pathogenic or probably pathogenic variants and one patient with a variant of unknown significance were identified in BaMaRa^®^ of whom 10 met non-inclusion criteria (8 underaged, 1 breastfeeding, 1 under guardianship), 4 declined and 5 could not be reached. Finally, we analyzed 10 women with a median age of 40.5 years (IQR: 33.0-49.5). Patients’ characteristics are reported in Table 1 with genetic data detailed in Table 2.

**Table 1:** Characteristics of patients in the whole population and in patients with emphysema

	Whole population (*n* = 10)	With emphysema (*n* = 7)
**Demographic features**		
Female	10 (100%)	7 (100%)
Age, years	40.5 (33.0;49.5)	42.0 (31.0;52.0)
BMI, kg/m^2^	27.6 (22.4;30.6)	27.1 (20.8;28.1)
**Medical history**		
Neonatal acute respiratory failure	2 (20%)	1 (14%)
Previously known emphysema*	3 (30%)	3 (43%)
Asthma	2 (20%)	0 (0.0%)
Pulmonary embolism	1 (10%)	1 (14%)
Hypertension	1 (10%)	1 (14%)
Supraventricular arrhythmia	2 (20%)	1 (14%)
Aortic root dilatation	3 (30%)	2 (29%)
Pulmonary hypertension	2 (20%)	2 (29%)
Congenital cardiopathy	2 (20%)	1 (14%)
Valvular disease	3 (30%)	1 (14%)
PVNH	5 (50%)	4 (57%)
Melnick-Needles syndrom	1 (10%)	1 (14%)
**Environmental exposure**		
Tobacco smoker > 5 pack-years	0 (0%)	0 (0%)
Tobacco passive smoker	4 (40%)	3 (43%)
Cannabis smoker	0 (0%)	0 (0%)
Household	0 (0%)	0 (0%)
Occupational	0 (0%)	0 (0%)
Air pollution	0 (0%)	0 (0%)
**Respiratory symptoms**		
Chronic cough	2 (20%)	0 (0%)
Exertional dyspnea	8 (80%)	5 (71%)
mMRC0	1 (10%)	0 (0%)
mMRC1	3 (30%)	1 (14%)
mMRC2	2 (20%)	2 (29%)
mMRC3	1 (10%)	1 (14%)
mMRC4	1 (10%)	1 (14%)
Chronic bronchitis	1 (10%)	0 (0%)
**CT scan**		
Centrilobular	-	4.0 (57%)
Panlobular	-	2.0 (29%)
Paraseptal	-	2.0 (29%)
Upper predominance	-	4.0 (57%)
No predominance	-	3.0 (43%)
Volume of emphysema, %	-	8.8 (3.0;18.0)
**Pulmonary function tests**		
FEV1, Z-score	-2.40 (-3.37;-1.44)	-1.78 (-3.51;-0.70)
FEV1 < LLN	7 (70%)	4 (57%)
FEV1/FVC, Z-score	-1.96 (-2.76;-1.14)	-1,29 (-2.39;-0.79)
FEV/FVC < LLN	6 (60%)	3 (43%)
FEF25-75, Z-score	-2.10 (-3.28;-0.32)	-1.28 (-2.44;0.32)
FEF25-75 < LLN	5 (50%)	2 (29%)
RV/TLC	2.01 (1.40;2.26)	2.00 (0.80;2.50)
RV/TLC > ULN	7 (70%)	4 (57%)
DLCO, Z-score	-1.56 (-2.68;-0.03)	-2.41 (-3.49;-1.41)
DLCO < LLN	5 (50%)	5 (71%)
R5, Z-score	1.43 (0.64;2.02)	0.77 (0.60;1.83)
R5 > ULN	4 (40%)	2 (29%)
X5, Z-score	1.57 (-0.13;2.47)	0.12 (-0.22;2.13)
X5 > ULN	3 (30%)	1 (14%)
AX, Z-score	1.72 (0.62;2.86)	0.97 (0.51;2.8)
AX > ULN	5 (50%)	2 (29%)
R5-R20, kPa/L/s	0.11 (0.08;0.20)	0.09 (0.08;0.17)
R5-R20 > 0.07	7 (70%)	5 (71%)

Values reported as n (%) for qualitative variables and median (IQR) for quantitative variables; IOS values (R5, R5-R20, X5, AX) were not available in 2 patients with emphysema

AX: integrated area of low frequency reactance; BMI: body mass index; DLCO: diffusion capacity for carbon monoxide; FEF25-75 : forced expired flow between 25 and 75% of FVC; FEV1 : forced expiratory volume 1 s; *FLNA* : filamin A; FVC : forced vital capacity; LLN: lower limit of normal; mMRC: modified Medical Research Council; PVNH: periventricular nodular heterotopia; R5: resistance at 5 Hz; RV: residual volume; TLC: total lung capacity; ULN: upper limit of normal; X5: reactance at 5 Hz; * : In all these cases, emphysema was diagnosed in adulthood

**Table 2:** Description of FLNA variants and related clinical features

*n*	HGVS DNA on transcript	HGVS Protein	Zygosity	ACMG class	Type	FLNA related features
*With emphysema*						
1^+^	c.730 C > T	p.(Pro244Ser)	Heterozygous	4	Missense	Melnick-Needles syndrome
2*	c.134 A > G	p.(Gln45Arg)	Heterozygous	4	Missense	PVNH, aortic root dilatation, valvular disease
3*^F^	c.7898_7900del	p.(Gly2633del)	Heterozygous	4	Inframe	
4^− F^	c.7609del	p.(Ser2537LeufsTer2)	Heterozygous	5	Frameshift	
5*	c.(?_1)_(2022_?)del	N.A	N.A	4	Deletion	PVNH, joint hypermobility, congenital heart disease, pulmonary hypertension, neonatal acute respiratory failure
6*	c.992_993del	p.Lys331SerfsTer5	Heterozygous	5	Frameshift	PVNH, dilatation of thoracic aorta
7*	c.992_993del	p.Lys331SerfsTer5	Heterozygous	5	Frameshift	PVNH, pulmonary hypertension
*Without emphysema*						
8^−^	c.7306G > A	p.(Gly2436Arg)	Heterozygous	3	Missense	PVNH, valvular disease, patent ductus arteriosus, neonatal acute respiratory failure, congenital cardiopathy
9^−^	c.1966 C > T	p.(Leu656Phe)	Heterozygous	4	Missense	Dilatation of thoracic aorta, valvular disease
10^− F^	c.7941_7942del	p.(Ter2648SerextTer100)	Heterozygous	4	Stop-loss	

ACMG : American College of Medical Genetics and Genomics. HGVS : Human Genome Variation Society. N.A : not available. PVNH : periventricular nodular heterotopia. Function of the protein : * : loss of function; + : gain of function; - : not available. ^F^ : genetic testing performed because of family history. These patients did not have clinical involvement suggestive of a FLNA mutation. All patients reported in Table 2 are women

The frequency of emphysema was 70.0%. It was most often centrilobular and located in upper lobes (*n* = 4) with a median volume of 8.8% (IQR: 3.0–18.0%) of the total lung volume. Among patients with emphysema, PFT showed airflow limitation in 3 cases, lung hyperinflation in 4 cases, reduced DLCO in 5 cases and SAD in all 5 emphysema patients who performed IOS, together with an increased AX in 2 of them (Table 1). Two patients without emphysema had asthma and presented reduced FEV1/FVC and FEV1, and SAD.

In the emphysema group, three patients reported significant passive tobacco smoking, and four met the criteria of unexplained emphysema. The frequency of unexplained emphysema was therefore estimated to be 57.1% of emphysema cases.

In this preliminary study, we found a significant frequency of emphysema estimated at 70% in patients with *FLNA* pathogenic or probably pathogenic variants among which 57.1% are unexplained. The volume of emphysema was clinically relevant as it represented 8.8% (IQR: 3.0–18.0%) of the total lung volume while it was reported to represent 1.2% (IQR: 0,5–2,5%) of the total lung volume in healthy non-smoker people [8]. The only patient with a gain-of-function mutation had minimal emphysema (0.2%) without exposure to tobacco. These data suggest that *FLNA* pathogenic variants, particularly those associated with loss-of-function may be associated with emphysema. This is supported by experimental data reporting emphysema in mice with loss-of-function *FLNA* pathogenic variants [9], possibly due to abnormal endothelial adherens junctions.

PFT revealed abnormalities usually found in patients with emphysema, the most frequent being reduced DLCO [2, 3]. They also revealed SAD in all emphysema patients who performed IOS, consistently with a previous study reporting a good diagnosis accuracy of IOS-diagnosed SAD for the detection of emphysema [10].

The main limit of this study is the small sample size, due to the scarcity of *FLNA* pathogenic variants and the monocentric design. We decided to carry out this pilot study on a limited number of patients to assess the relevance of our hypothesis, given the irradiating nature of chest CT.

In conclusion, our results suggest that adult women with loss-of-function *FLNA* pathogenic variants develop emphysema. They encourage focusing on measures for lung protection and paying particular attention on respiratory symptoms in this condition.

## Data Availability

The data that support the findings of this study are available on request from the corresponding author. The data are not publicly available due to privacy or ethical restrictions.

## Declarations

### Notation of prior abstract publication/presentation

Abstract in a French congress : CPLF 2025 (24-26th January 2025 in Marseille) and in the ERS congress 2025 (27 September to 1 October 2025 in Amsterdam).

### “Take home” message

Emphysema seems frequent in adult women with *FLNA* pathogenic variant encouraging a systematic screening and measures for lung protection including avoidance of passive smoking.

## Abbreviations

AAT: Alpha1antitrypsin deficiency
AX: Integrated area of low frequency reactance
DLCO: Diffusion capacity for carbon monoxide
FEF25-75: Forced expiratory flow between 25 and 75% of FVC
FEV1: Forced expiratory volume in 1 s
FLNA: Filamin A gene
FVC: Forced vital capacity
GLI: Global lung initiative
IOS: Impulse oscillometry
IQR: Interquartile range
PFT: Pulmonary function tests
PVNH: Periventricular nodular heterotopia
R5: Resistance at 5 Hz
RV: Residual volume
SAD: Small airway dysfunction
TLC: Total lung capacity
X5: Reactance at 5 Hz

## References

[1] Sasaki E, Byrne AT, Phelan E, et al. A review of filamin A mutations and associated interstitial lung disease. Eur J Pediatr. 2019;178(2). 10.1007/s00431-018-3301-0. 10.1007/s00431-018-3301-0 30547349

[2] Kremer TM, Lindsay ME, Kinane TB, et al. Case 28-2019: A 22-Year-Old woman with dyspnea and chest pain. N Engl J Med. 2019;381(11):1059–67. 10.1056/NEJMcpc1904041. 31509678 10.1056/NEJMcpc1904041

[3] Tanner LM, Kunishima S, Lehtinen E, et al. Platelet function and filamin A expression in two families with novel FLNA gene mutations associated with periventricular nodular heterotopia and panlobular emphysema. Am J Med Genet A. 2022;188(6):1716–22. 10.1002/ajmg.a.62690. 35156755 10.1002/ajmg.a.62690 PMC9303863

[4] Lange M, Kasper B, Bohring A, et al. 47 patients with FLNA associated periventricular nodular heterotopia. Orphanet J Rare Dis. 2015;10(1):134. 10.1186/s13023-015-0331-9. 26471271 10.1186/s13023-015-0331-9 PMC4608144

[5] Valentin V, Bervar JF, Vincent-Delorme C, et al. Filamin A mutations: A new cause of unexplained emphysema in adults? Chest. 2021;159(3):e131–5. 10.1016/j.chest.2020.10.003. 33678279 10.1016/j.chest.2020.10.003

[6] Oostveen E, Boda K, Van Der Grinten CPM, et al. Respiratory impedance in healthy subjects: baseline values and bronchodilator response. Eur Respir J. 2013;42(6):1513–23. 10.1183/09031936.00126212. 23598954 10.1183/09031936.00126212

[7] Bossé Y, Lamontagne M, Gaudreault N, et al. Early-onset emphysema in a large French-Canadian family: a genetic investigation. Lancet Respir Med. 2019;7(5):427–36. 10.1016/S2213-2600(19)30056-6. 31000475 10.1016/S2213-2600(19)30056-6

[8] Hoffman EA, Ahmed FS, Baumhauer H, et al. Variation in the percent of Emphysema-like lung in a Healthy, nonsmoking multiethnic Sample. The MESA lung study. Ann Am Thorac Soc. 2014;11(6):898–907. 10.1513/AnnalsATS.201310-364OC. 24983825 10.1513/AnnalsATS.201310-364OC PMC4213995

[9] Feng Y, Chen MH, Moskowitz IP, et al. Filamin A (FLNA) is required for cell–cell contact in vascular development and cardiac morphogenesis. Proc Natl Acad Sci U S A. 2006;103(52):19836–41. 10.1073/pnas.0609628104. 17172441 10.1073/pnas.0609628104 PMC1702530

[10] Klitgaard A, Løkke A, Hilberg O. Impulse oscillometry as a diagnostic test for pulmonary emphysema in a clinical setting. J Clin Med. 2023;12(4):1547. 10.3390/jcm12041547. 36836082 10.3390/jcm12041547 PMC9967696

